# Genome-Wide Functional Analysis of Cotton (*Gossypium hirsutum*) in Response to Drought

**DOI:** 10.1371/journal.pone.0080879

**Published:** 2013-11-18

**Authors:** Yun Chen, Zhi-Hao Liu, Li Feng, Yong Zheng, Deng-Di Li, Xue-Bao Li

**Affiliations:** Hubei Key Laboratory of Genetic Regulation and Integrative Biology, College of Life Sciences, Central China Normal University, Wuhan, China; Wuhan University, China

## Abstract

Cotton is one of the most important crops for its natural textile fibers in the world. However, it often suffered from drought stress during its growth and development, resulting in a drastic reduction in cotton productivity. Therefore, study on molecular mechanism of cotton drought-tolerance is very important for increasing cotton production. To investigate molecular mechanism of cotton drought-resistance, we employed RNA-Seq technology to identify differentially expressed genes in the leaves of two different cultivars (drought-resistant cultivar J-13 and drought-sensitive cultivar Lu-6) of cotton. The results indicated that there are about 13.38% to 18.75% of all the unigenes differentially expressed in drought-resistant sample and drought-sensitive control, and the number of differentially expressed genes was increased along with prolonged drought treatment. DEG (differentially expression gene) analysis showed that the normal biophysical profiles of cotton (cultivar J-13) were affected by drought stress, and some cellular metabolic processes (including photosynthesis) were inhibited in cotton under drought conditions. Furthermore, the experimental data revealed that there were significant differences in expression levels of the genes related to abscisic acid signaling, ethylene signaling and jasmonic acid signaling pathways between drought-resistant cultivar J-13 and drought-sensitive cultivar Lu-6, implying that these signaling pathways may participate in cotton response and tolerance to drought stress.

## Introduction

Drought is one of the most important abiotic stresses affecting plant growth in the world. It often impacts the physiological and metabolic processes of plants, while plants have also formed various mechanisms to cope with it. These mechanisms have been classified into three groups: drought escape, drought avoidance and drought tolerance [1]. Drought escape allows plants finish their life cycles during the period of sufficient water supply. To most important economic or food crops, however, it is impracticality for people to change their life cycles. Drought avoidance helps plants maintain high water status during periods of stress by enhancing water absorption or/and reducing evapotranspiration, while drought tolerance means plants maintain turgor and continue metabolism in cells even at low water potential, mainly by protoplasmic tolerance, synthesis of osmolytes or/and compatible solutes [2]. Both drought avoidance and drought tolerance can be used in the improvement of crops.

Upland cotton (*Gossypium hirsutum*) is one of the most important crops planted widely in China for its natural textile fibers. Upland cotton has a complex allotetraploid genome (AADD), with a haploid genome size estimated to be around 2.5 Gb [3,4]. In the most regions of China, cotton is planted during the period of April to October. Due to the uneven distribution of water resource in the seasons of cotton growth, breeders are mainly making efforts to enhance the drought avoidance and/or tolerance ability of cotton. There are two general efforts towards more sustainable and water-use-efficient agricultural production. One effort is focused on the improvement of agricultural technology [5,6], and another is represented by breeding and genetic engineering [7-9]. Breeding has been used to improve the drought tolerance of cotton, but the process is slow and limited [10]. Therefore, people tried to improve the drought tolerance of plants (such as cotton) by genetic engineering in recent years. It has been reported that overexpression of *GhZFP1*, *GhMPK2* and *GhMKK5* in tobacco affected the transgenic plant tolerance to salt and drought stress [11-13], while *GhDREB* and *GhMPK16* altered the tolerance to drought stress in transgenic wheat and *Arabidopsis*, respectively [14,15]. High throughout sequencing has also been used to search for drought-related genes in cotton along with the improvement of sequencing technology and the completion of diploid cotton genome sequencing [16-20]. Ranjan et al (2012) analyzed the drought-resistibility of different diploid cotton species, and found that the drought tolerance observed in Vagad is due to several mechanisms working together [18]. Park et al (2012) detected differentially expressed transcription from water deficit stressed root and leaf tissues in tetraploid cotton using cDNA-AFLP [21]. In this study, we analyzed the drought-resistibility of several cultivars of tetraploid upland cotton, and found that the cultivar Jingmian 13 (J-13) is more drought-resistible than Lumian 6 (Lu-6). In addition, we measured several physiological parameters (such as malondialdehyde, peroxidase, catalase and electrolyte leakage) for evaluating the difference in drought-resistance between J-13 and Lu-6. In order to discover the mechanism of cotton drought resistance, we used RNA-Seq to identify differentially expressed genes in the leaves of both J-13 and Lu-6 plants grown in drought-stress environment. 

## Results

### Verification of drought-tolerance of cotton cultivars

**Figure 1:**
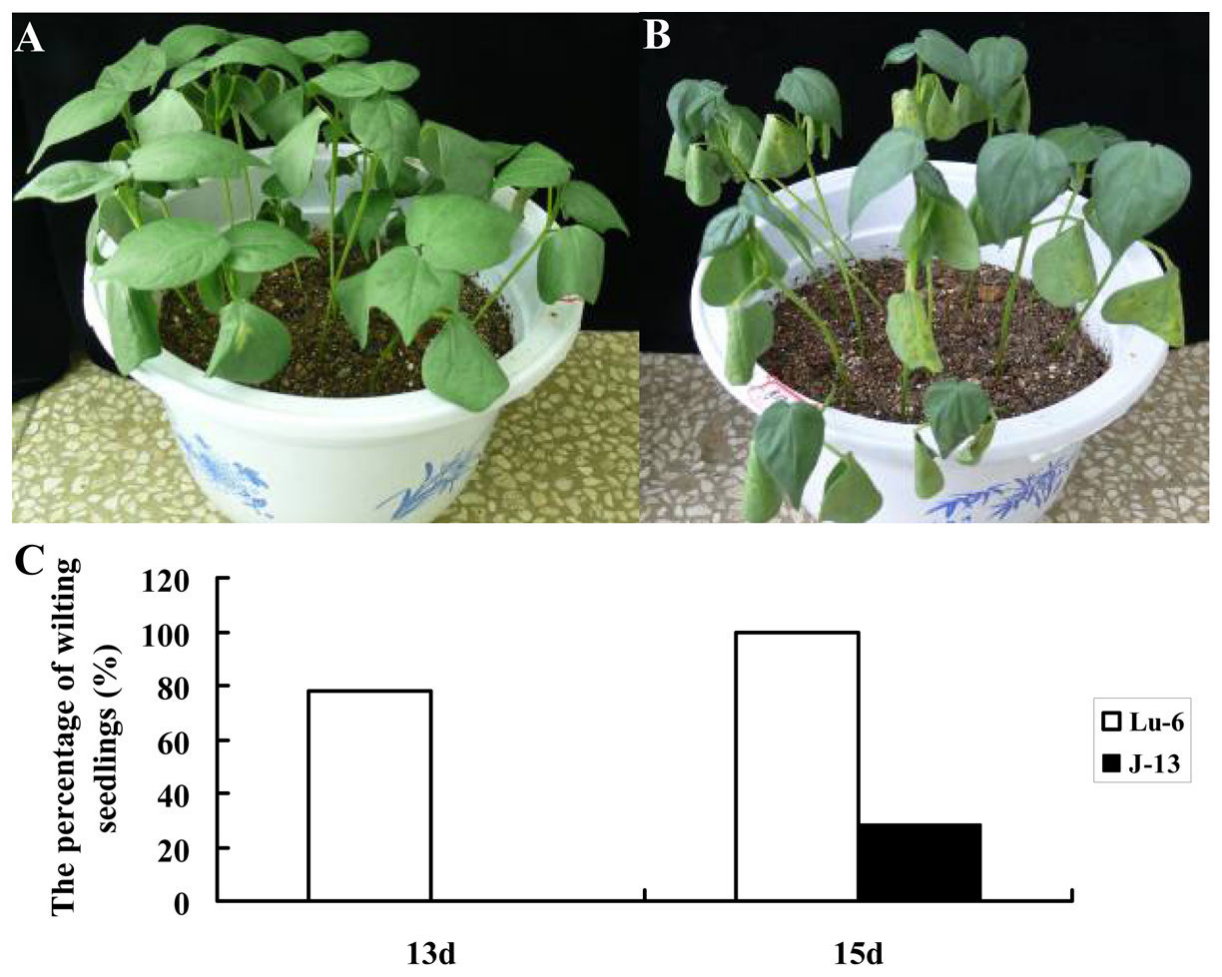
Assay of drought resistance of the two cotton cultivars. (**A**) Seedlings of the cultivar J-13 in pot after withholding water for 15 days. (**B**) Seedlings of the cultivar Lu-6 in pot after withholding water for 15 days. (**C**) The percentage of wilting seedlings after withholding water for 13 and 15 days. J-13, cotton cultivar Jingmian13; Lu-6, cotton cultivar Lumian6.

It has been reported that cotton cultivar Lu-6 is sensitive to drought stress, while the cultivar J-13 is tolerant to drought stress [27]. To test and verify the drought tolerance of Lu-6 (susceptible) and J-13 (resistant), we counted off 45 seeds of each cultivar and cultured them in sterilized soil (a complex of vermiculite and garden soil). The seedlings of each cultivar were grown in 3 pots (one pot as control and two pots for drought treatment). Control pots were irrigated every day, while drought-treated pots were withholding water, observing and counting the seedlings that had becoming wilting under dehydration condition. The experimental results indicated that no wilting seedling of J-13 was found, but 21 wilting seedlings of Lu-6 were observed after 13 days of water withholding. With the prolonged drought duration, only 8 seedlings of J-13 became slightly wilting, whereas all of the Lu-6 seedlings displayed severe wilting after 15 days of drought treatment (Figure 1). These results indicated that J-13 is more tolerant to drought stress than Lu-6.

### Comparison of several physiological parameters between J-13 and Lu-6 in response to drought stress

**Figure 2:**
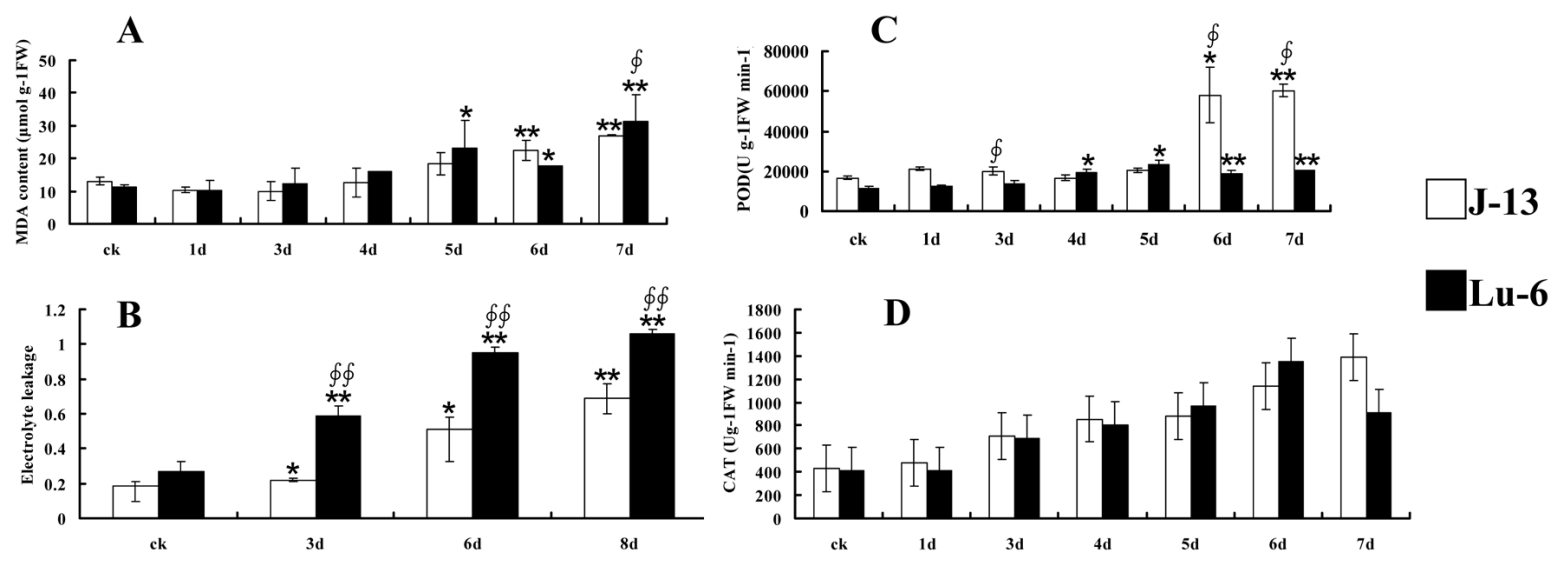
Effects of drought stress on lipid perxidation, electronlyte leakage, POD and CAT activity in the leaves of cotton cultivars J-13 and Lu-6. All the values are mean ±S.D. Independent *t*-tests demonstrated that there was significant (* *p*<0.05) or very significant (** *p*<0.01) difference between the drought-treated samples and controls, and there was significant (^∮^ *p*<0.05) or very significant (^∮∮^ *p*<0.01) difference between J-13 and Lu-6 at the same drought stress time point. Error bars were calculated from three independent experiments.

It has been reported that various physiological parameters will be changed in plant response to drought stress. Levels of malondialdehyde (MDA) and electrolyte leakage in cotton tissues were detected for evaluating the extent of cellular damage of both Lu-6 and J-13 during the drought treatment. As shown in Figure 2A, with the increase of drought treatment time, MDA contents of the two cultivars were gradually increased. Compared with the control, there was a significant increase in MDA content of Lu-6 plants after 4 days of drought treatment, and this increase became very significant after 7 days. In J-13 plants, MDA content was very significant increase after 5 days of drought treatment. When withholding water for 7 days, the content of MDA in Lu-6 plants was significant higher than that of J-13. We also detected the electrolyte leakage of the two cultivars under drought stress. Similarly, the levels of electrolyte leakage of both Lu-6 and J-13 were increased gradually during drought treatment (Figure 2B). However, there were very significant differences in the level of electrolyte leakage between Lu-6 and J-13 after 3 days of drought treatment. In addition, both peroxidase (POD) and catalase (CAT) activities were increased gradually under the drought treatment (Figure 2C and 2D). Interestingly, we found that POD activity in Lu-6 plants was significantly lower than that of J-13, although it was still increased along with the drought treatment. These results suggested that J-13 is more tolerance to drought than Lu-6 owing to the difference in physiological metabolisms in cells between J-13 and Lu-6 under drought stress. 

### Analysis of RNA-seq data

**Figure 3:**
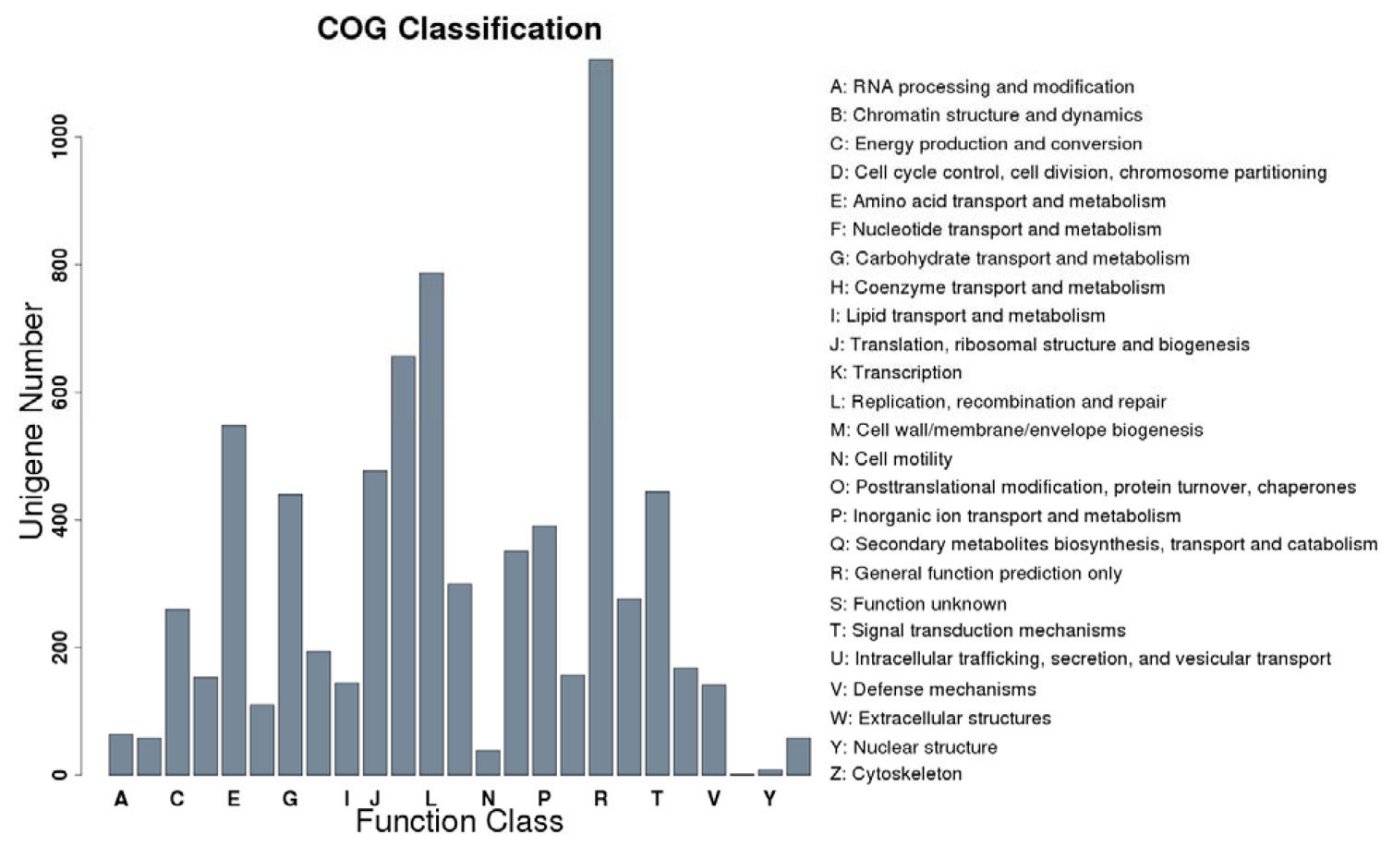
Histogram representation of clusters of orthologous groups (COG) classification.

**Figure 4:**
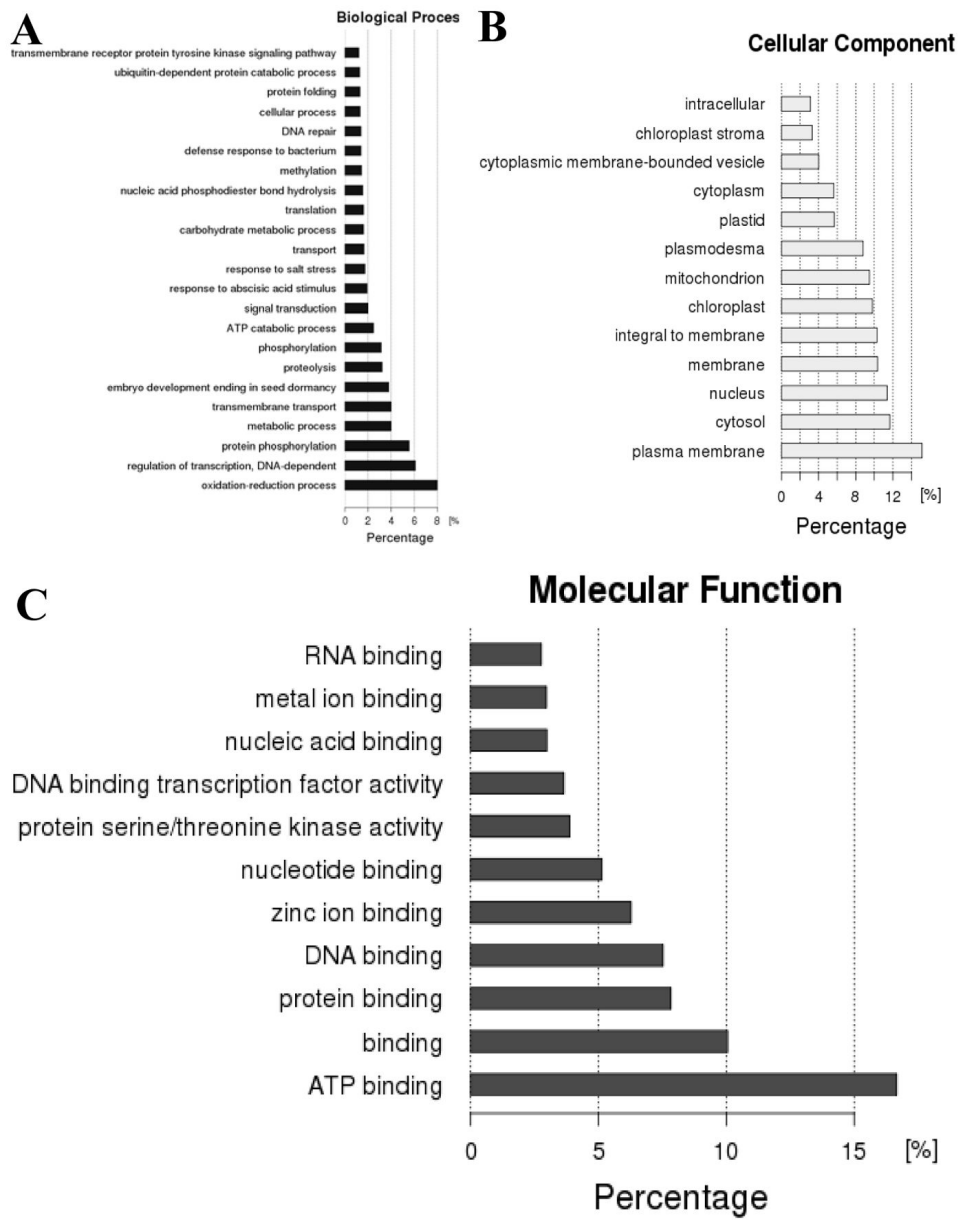
The percentage of each GO term in the GO classification. (**A**) The Biological Process percentage. (**B**) The Cellular Component percentage. (**C**) The Molecular Function percentage.

For each sample, there were about 15 million clean reads (Table 1), and a total of 77,231 fragments were generated. These fragments were analyzed whether to match to the reported cotton contigs in GenBank databases by blastn (E-value≤1e-5). Among them, 43,907 unigenes could be mapped to the reported unigenes, and the remaining 33,324 ones did not matched to any reported ones in cotton databases. We further matched these 33,324 unigenes to different databases, and the results suggested the matching rate was from 19.68% to 61.61% (Table 2). The number of unigenes that mapped to Nr database was the most (61.61%) while mapped to COG database was the least (19.68%). These results implied that parts of the 33,324 unigenes have not been collected in the Nr database in transcriptome research. COG database mainly describes the clustering of ortholog proteins from both prokaryote and single-celled eukaryote. We performed cluster analysis of 6,559 genes that could mapped to COG, and found that these genes were enriched in amino acid transport and metabolism, carbohydrate transport and metabolism, transcription, general function prediction only, and replication, recombination and repair (Figure 3). GO-based classification was conducted and blast-go was used to GO annotation. A total of 13,598 genes were annotated from 33,324 new unigenes, and a total of 3,903 GO terms participated in their annotation. Analysis of categories revealed that these new genes were classified into 23 biological processes, such as response to salt stress, abscisic acid (ABA), signal transduction, transmembrane transport, protein phosphorylation, oxidation-reduction process (Figure 4A). These genes were distributed in membrane, nucleus and plastid (Figure 4B), and they mainly perform 11 functions (including DNA binding, metal ion binding, protein binding, and so on) in cotton (Figure 4C).

**Table 1:** The high-quality RNA-seq reads number of six different samples.

**Sample**	**Clean reads**
J13-ck	12,153,267
J13-2d	11,939,375
J13-4d	13,713,897
J13-6d	18,877,592
Lu6-6d	16,447,331
J13-8d	17,776,639

J13-ck, J13-2d, J13-4d, J13-6d and J13-8d refer to control (J13 plants grown under normal conditions) and 2, 4, 6, and 8 days after withholding water of J-13. Lu 6-6d refers to 6 days after withholding water of Lu-6.

**Table 2:** Statistical analysis of blast results of cotton unigenes identified in RNA-seq libraries.

**Database**	**Total Unigenes**	**Mapped Unigenes**
**COG**	33324	6559 (19.68%)
**Nr**	33324	20532 (61.61%)
**Nt**	33324	17543 (52.64%)
**Swissprot**	33324	12650 (37.96%)
**Trembl**	33324	20449 (61.36%)

All 33324 new unigenes were mapped to five databases.

### Identification of the differentially expressed genes in cotton under different conditions

**Figure 5:**
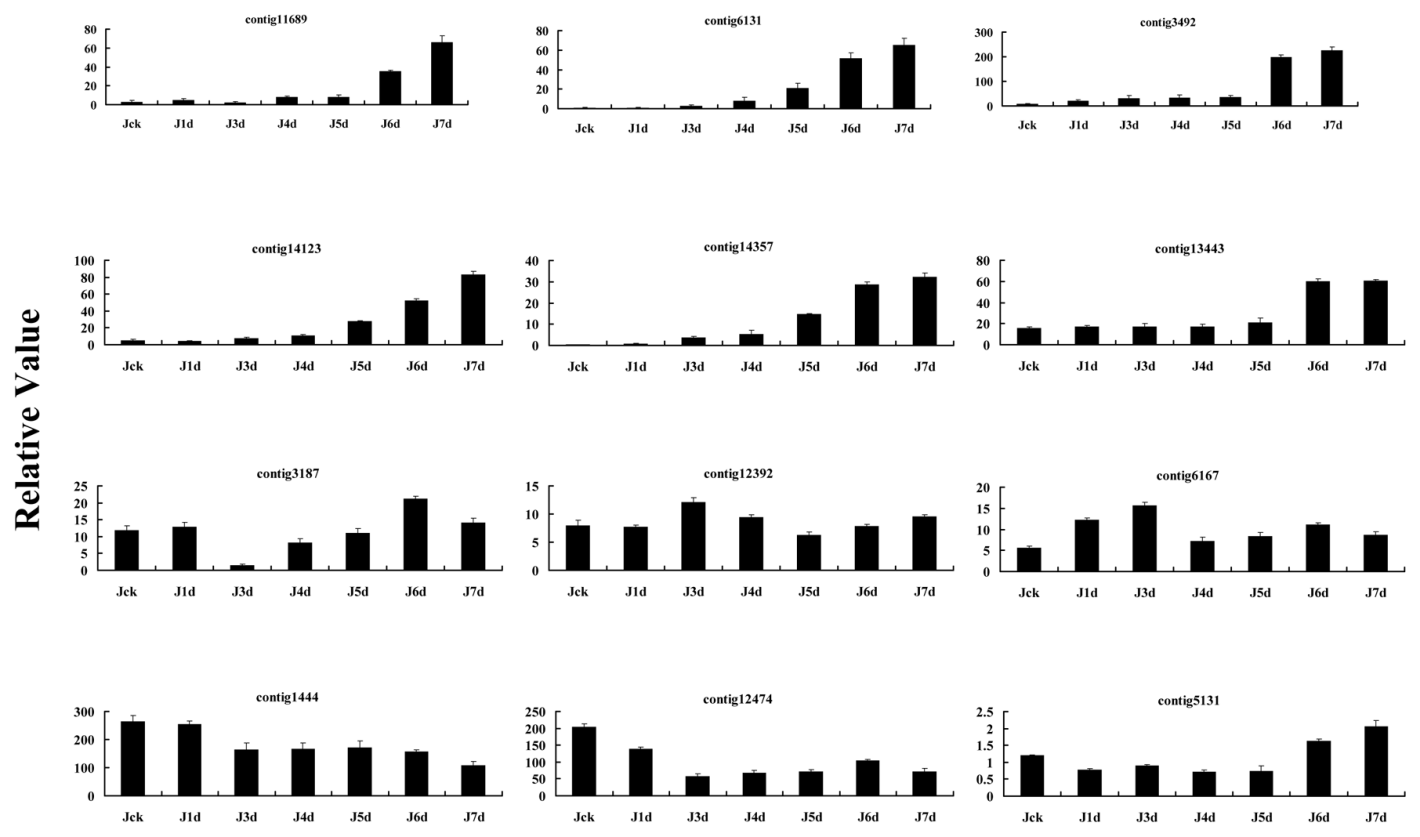
Expression profiles of the isolated genes in leaves of cotton J-13 seedlings under drought stress. Relative expression levels were determined by real-time quantitative RT-PCR for data verification. Error bars represent the SD for three independent experiments, and three technical replicates were analyzed. Jck, J1d, J3d, J4d, J5d, J6d and J7d on the x-axis refer to cotton J-13 seedlings after withholding water for 0 (control), 1, 3, 4, 5, 6, and 7 days. The y-axis represents the relative expression level.

To validate the results of the RNA-seq data, 12 genes, which included up-regulated, unchanged, and down-regulated genes identified through RNA-seq analysis, were analyzed using real-time RT-PCR (Figure 5). The results of this experiment were basically consistent with RNA-seq data.

**Figure 6:**
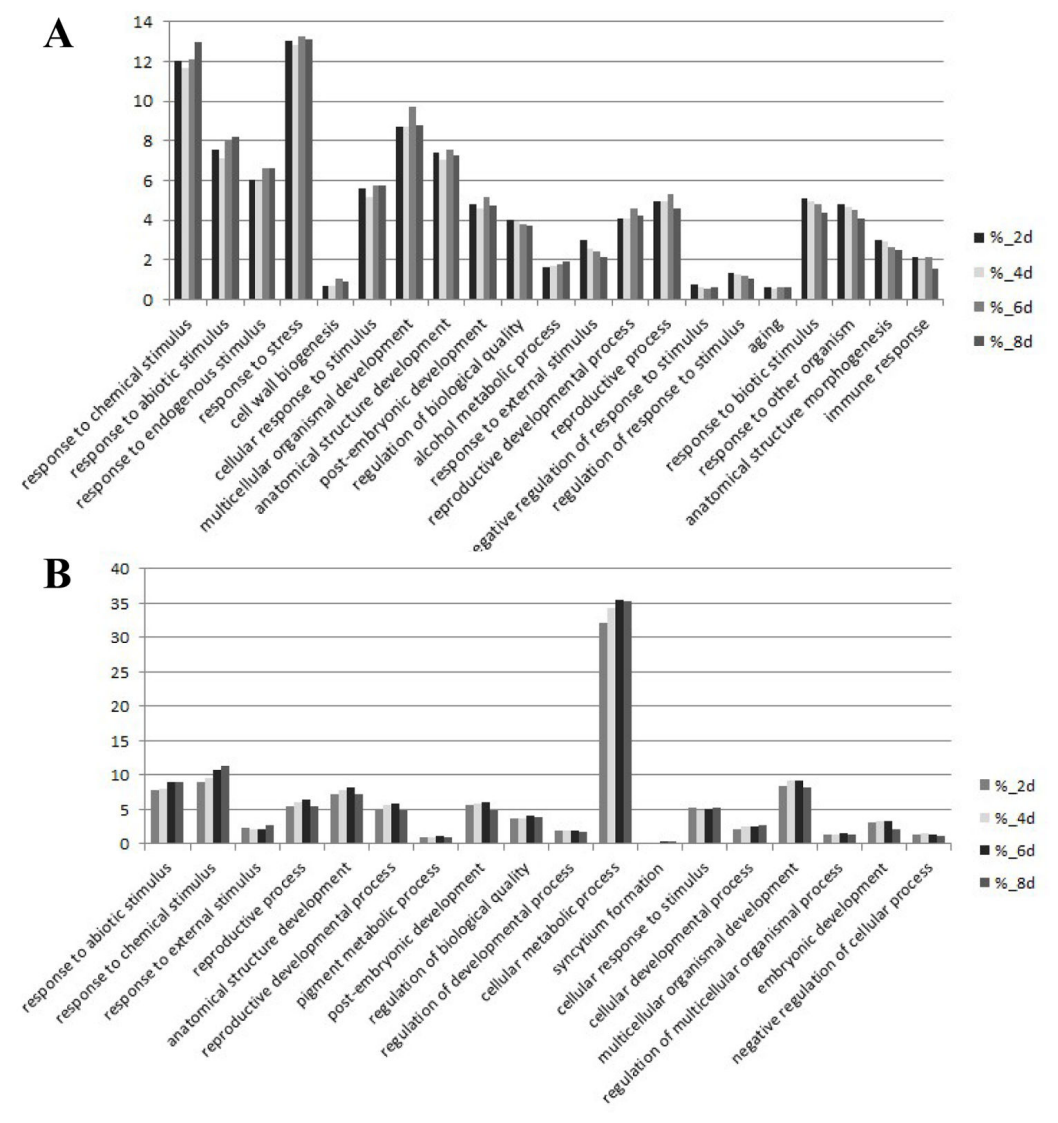
GO functional classification of cotton J-13. GO functional classification analysis between control and 2, 4, 6 and 8 day-drought samples. Histograms represent the functional distribution, which is expressed as a percentage of the amount of genes.

**Figure 7:**
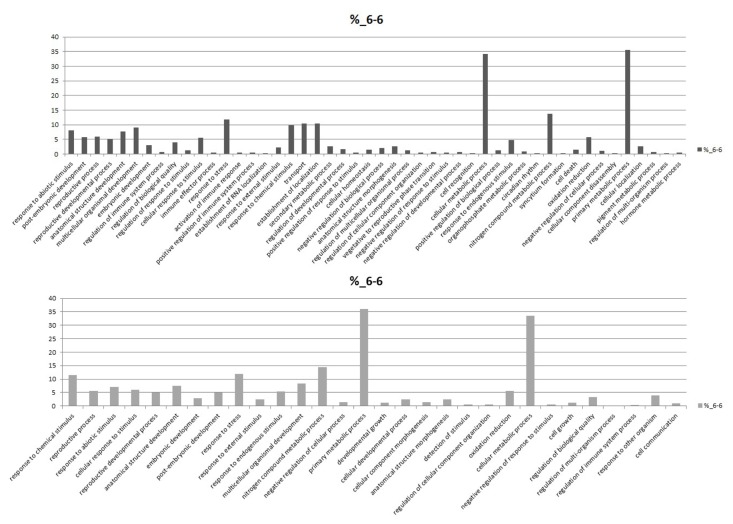
GO functional classification of two different samples. GO functional classification analysis of differentially expressed genes between J-13 and Lu-6 after 6 days of drought treatment. Histograms represent the functional distribution, which is expressed as a percentage of the amount of genes.

We combined the reported cotton contigs with the new unigenes found in this RNA-seq assay as a new cotton gene database, and mapped all clean reads to it. Subsequently, we noted these genes with *Arabidopsis* genes and analyze different expressed genes by edger program. The statistical results showed that the percentage of different expressed genes was between 13.38% and 18.75% (Table 3). Compared with the control sample, the number of different expressed genes of J-13 (a drought-resistant cultivar of cotton) was gradually increased with the drought stress time increasing. There was also great difference in expression levels of these genes between J-13 and Lu-6 after withholding water for 6 days. Furthermore, we analyzed the differential expression genes by DAVID (http://david.abcc.ncifcrf.gov/tools.jsp). As shown in Figure 6, over 30% of the down-regulated genes in J-13 during drought treatment were involved in cellular metabolic process and most of these down-regulation genes are chloroplast-/plastid-related, while the rest down-regulated genes were mainly distributed through the response to abiotic and chemical stimulus, anatomical structure development and multicellular organismal development. On the other hand, the genes mainly up-regulated during the drought treatment include the genes involved in response to chemical, abiotic stimulus, biotic stimulus, endogenous stimulus and stress. These results implied that the inhibition of cellular metabolic process (mainly photosynthesis) may be one of the response mechanisms in J-13. Compared with Lu-6, the genes involved in abiotic stimulus, radiation, light stimulus, defense response to bacterium and response to abscisic acid stimulus were up-regulated after drought treatment for 6 days. However, the genes involved in ethylene response, jasmonic acid biosynthetic and metabolic process were down-regulated in J-13 (Figure 7 and Table S1). 

**Table 3:** Total number of differentially expressed unigenes (fold change≥2 or ≤0.5).

**DEG sample**	**Total unigene**	**P-value**	**Down regulated unigene NO.**	**Up regulated unigene NO.**	**Total**
J13-2d vs J13-ck	61756	0.01	4630	5344	9974
J13-4d vs J13-ck	61756	0.01	5705	5875	11580
J13-6d vs J13-ck	61756	0.01	3227	5036	8263
J13-8d vs J13-ck	61756	0.01	4094	5493	10397
J13-6d vs Lu 6-6d	61756	0.01	5067	5808	10875

J13-ck, J13-2d, J13-4d, J13-6d and J13-8d refer to control (J13 plants grown under normal conditions) and 2, 4, 6, and 8 days after withholding water of J-13. Lu 6-6d refers to 6 days after withholding water of Lu-6.

### Searching of continuously up-regulated and down-regulated genes in J-13 response to drought stress

We analyzed the gene expression profiling in five J-13 samples, and found that there were 229 continuously down-regulated (Co-Down) and 627 continuously up-regulated (Co-Up) genes (Table 4). The Co-Up genes were assigned to cell wall synthesis, response to water deprivation, response to abscisic acid stumulus and so on, while genes related to ethylene signaling pathway were continuously down-regulated (Table S2). These results suggested that a lot of genes are expressed temporally and specifically in plant response to drought treatment, and the abscisic acid signaling is continually activated in plants during the whole drought process. 

**Table 4:** Continually-differentially expressed unigenes at different drought period.

**Group**	**Down-regulation**	**Co-down-regulation**	**Up-regulation**	**Co-up-regulation**
J13-2d vs J13-ck	2223		2649	
J13-4d vs J13-ck	2315	229	2762	627
J13-6d vs J13-ck	1022		2055	
J13-8d vs J13-ck	2511		2785	

J13-ck, J13-2d, J13-4d, J13-6d and J13-8d refer to control (J13 plants grown under normal conditions) and 2, 4, 6, and 8 days after withholding water of J-13.

## Discussion

Cotton is one of the most important economic crops and is planted all over the world [28,29]. Although there are more than 50 species in the genus *Gossypium*, only four of them are cultivated in the world. Upland cotton (*Gossypium hirsutum* L.) is the most widely planted, accounting for more than 95% of the annual cotton crop worldwide. Upland cotton is an allotetraploid species with a large and complex genome (nearly 2.5G). Recently, Ranjan has analyzed the drought tolerance mechanisms in diploid cotton by Gene chip [18], while Park detected differentially expressed genes in tetraploid cotton under water deficit [21]. Their results revealed some drought-regulated genes, but can not display the changes of gene expression in cotton species during the whole drought response process. In this study, we analyzed the continuous changes of gene expression in upland cotton (J-13) during drought treatment, and compared the differences between J-13 (drought-tolerant cultivar) and Lu-6 (drought-sensitive cultivar). Our data provide more information for us to understand the mechanisms of cotton response to drought stress.

Phenotype observation and physiological parameter measurement suggested that J-13 has better physiological status than Lu-6 during drought treatment. Samples harvested from different time points were sequenced using RNA-Seq, respectively. Although the completion of diploid cotton genome sequencing provides useful information for us, it is not enough for researches based on tetraploid cotton. In order to get more information, we assembled the sequences from six samples together and analyzed the gene expression patterns individually. A total of 77,231 fragments were generated after assembled, and 43,907 of them could be mapped to the reported unigenes, while the remaining 33,324 fragments were matched to different databases, 13,598 of which were annotated, providing useful information for our future research. Furthermore, a group of selected genes with different expression patterns were analyzed by RT-PCR, and the results were basically consistent with RNA-seq, indicating that our RNA-seq data is credible. Analysis of categories revealed that the differentially expressed genes were classified into some biological processes, including metabolism, transport, defense response, protein modification, hormone response, and so on, suggesting that normal developmental processes were strongly affected in cotton plants treated with drought.

In the up-regulation gene clusters, the genes related to plant cell wall synthesis, defense response and protein modification always appeared in the top ten during the whole drought treatment. The genes encode peroxidases and related to phenylpropanoid biosynthesis pathways were up-regulated in J-13 under drought stress. Phenylpropanoid compounds belong to plant secondary metabolites, and may be involved in protecting plants from many abiotic and biotic stresses [30-32]. These up-regulated expression activities should enhance plant resistance to oxidative damage. Protein modification is very important to protein function or stability. Recently, it has been reported that both phosphorylation and dephosphorylation of ABI5 are important for regulation of ABA signaling [33,34]. Genes involved in the protein modification process may be important for regulating plant growth and development. After drought treatment for 4 days, the genes response to abscisic acid stimulus enrich in the up-regulation gene clusters and the function of ABA in stress response is proverbial [35]. After drought treatment for eight days, there was a high expression and enrichment of the genes involved in plant response to drought, osmotic and salt stresses. As many as 179 genes appeared in the first cluster, while these genes did not enrich in the sample without drought treatment. It may imply that cotton J-13 was in a state of hydropenia after 8 days of drought stress, and the osmotic and salt stress responses were activated in plants. 

During the whole drought treatment process, chloroplast-, plastid-, thylakoid- and photosynthesis-related genes were enrichment in the down-regulated gene cluster, suggesting that the photosynthesis was reduced in J-13 plants under drought conditions. Furthermore, the genes related to cell wall synthesis, external encapsulating structure, response to water deprivation, response to abiotic stimulus, response to abscisic acid stimulus, defense response to bacterium, lignin metabolic process, secondary metabolic process, nucleotide-sugar metabolic process and so on were up-regulated, suggesting that these genes may be important for cotton resistance to drought. When these up-regulated genes were matched to the KEGG database, it was found that these genes may be involved in the following pathways, including amino sugar and nucleotide sugar metabolism, phenylpropanoid biosynthesis, galactose metabolism, arginine and proline metabolism. These pathways may be important for cotton resistant to drought stress. It has been known that proline accumulates in plants in response to different stresses, including drought and high salinity, and protects cells against stress damage [36-39]. So, cotton J-13 may respond to drought by increase cellar proline content. 

Water deficit can induce plant wilting, damage to cell membranes and ultimately cell death when plants continuously lose water [40]. Some drought-tolerant plant species can tolerate or avoid drought stress by promoting stomatal closure, reducing leaf and stem growth, maintaining/increasing root extension [41], and/or increasing root and shoot hydraulic conductance [42,43]. After withheld water for 6 days, the genes involved in response to abscisic acid stimulus were expressed at higher levels in J-13, while the genes related to ethylene signaling pathway and jasmonic acid biosynthetic process were expressed at lower levels in J-13, compared with those in Lu-6. Previous studies provide the evidence for ABA-induced reduction of leaf growth rate and stomatal closure when ABA is generated endogenously via soil drying, and then plant tolerance to drought is increased. And it was reported that various stresses induce the production of ethylene which induces leaf senescence [44], and the stomata response to ABA is suppressed as leaves senesce [45]. In addition, to achieve precisely regulating defense responses, salicylic acid, ethylene and jasmonic acid (JA) signaling pathways may promote each other, or be antagonistic [46,47]. These factors may lead to the difference in drought tolerance between cotton cultivars J-13 and Lu-6.

## Materials and Methods

### Plant materials under drought treatment

Two different cultivars Jingmian 13 (J-13) and Lumian 6 (Lu-6) of upland cotton (*Gossypium hirsutum* L.) were used as experimental materials. Cotton seeds were sown in 12 pots (garden soil) in a controlled environmental growth chamber (14 h light/10 h dark, 28°C and 50 – 60% relative humidity). Drought treatment of the seedlings was initiated at three-leaf stage by withholding water, and leaf samples were collected from both well-watered and stressed seedlings by withholding water for one to seven days. Leaf samples were immediately crushed in liquid nitrogen and kept in –80°C till further analysis.

### Protein extraction and measurement of enzyme activity

Leaf samples (0.5 g) were thoroughly ground to powder in liquid nitrogen. One ml of ice-cold 150 mM potassium phosphate buffer (pH 7.0) was added and mixed with the samples for several minutes, and then 3 ml extraction solution was added to the mixture. The homogenates were centrifuged at 15,000 g for 20 min at 4°C. Supernates were collected for assaying enzyme activities and other physiological parameters. 

Peroxidase (POD) activity was measured by detecting the increase in absorbency at 460 nm as guaiacol was oxidized, according to the method of Chance and Maehly [22]. 50 μl enzyme extraction was added to the 2.85 ml of reaction mixture consisting of 1.85 ml 0.1M HAC-NaAC buffer (PH5.0), 0.25% guaiacol and 0.1ml 0.75% H_2_O_2_.

Catalase (CAT) activity was assayed by measuring the decomposing rate of H_2_O_2_ in absorbance at 240 nm [23]. The reaction solution (3 ml) contains 50 mM phosphate buffer (pH 7.0), 45 mM H_2_O_2_ and 100 μl enzyme extraction. Catalase activity was calculated by using extinction coefficient 39.4 mM^-1^ cm^-1^. 

### Measurement of lipid peroxidation

Malondialdehyde (MDA) is one of the most important membrane lipid peroxidation metabolites. In order to understand the degree of cotton leaf membrane lipid peroxidation under drought condition, we measured MDA content as described by Heath and Packer [24]. In brief, 1 ml sample solution (supernate as above) was extracted in 2 ml of a solution containing 0.25% TBA (Thiobarbituric acid) and 10% TCA (Trichloroacetic acid). The extraction was heated in a water bath at 95°C for 30 min and then quickly cooled in ice-water bath to room temperature. After centrifugation at 12,000 rpm for 15 min, the absorbance of the supernatant was measured at 532 nm and 600 nm (to eliminate the interference of non-specific impurities), respectively. The amount of MDA was calculated, based on adjusting absorbance and extinction coefficient 155 mM^-1^ cm^-1^.

### Electrolyte leakage

The degree of cell membrane injury induced by stress may be easily estimated through measurements of electrolyte leakage (EL) from the cells [25]. To determine electrolyte leakage, about 0.1 g leaves from every sample were excised and washed three times with deionized water. The washed leaves were cut to about 0.5 cm long pieces, placed in test tubes filled with 15 ml deionized water, and shaken for 24 h at room temperature. The initial conductivity (Ci) was determined using a conductivity meter (JENCO-3173, Jenco Instruments, Inc., San Diego, CA, USA). The test tubes were capped and autoclaved at 121°C for 30 minutes to completely disrupt the tissues and release all electrolytes. The conductivity (C_max_) of the incubation solution with killed tissues was determined after the solution cooled to room temperature. Relative EL was calculated using the formula: EL (%) = (Ci/Cmax)×100.

### RNA isolation and construction of cotton RNA-seq library

Two cotton cultivars (Lu-6 and J-13) were used for this study. For drought stress, plants were treated by withholding water. The control plants were irrigated daily. A total of sixty plants were grown in twenty flowerpots (thirty plants per cultivar, and three plants in each pot). Drought treatment was given to plants by stopped watering in 16 pots from both cultivars on three-leaf stage of the plants. The drought treatment was given till the visible differences became apparent. Remaining four pots from both cultivars were watered normally (controls). Thus, three plants from each cultivar in each pot at given condition were considered as biological replicates. After the drought treatment, samples were collected from plants withheld water one to eight days, respectively. The first leaves of all plants were collected for RNA extraction. Total RNA of leaf tissues was extracted by Spectrum plant total RNA Kit (Sigma-Aldrich) according to the manufacturer’s instructions. After DNase I treatment, RNA was purified by QIAquick PCR purification kit (Qiagen). Quality and quantity of the purified RNA were determined by measuring the absorbance at 260/280nm (A260/A280). RNA integrity was further verified by 1.5% agrose gel electrophoresis.

The six RNA samples, including five samples isolated from J-13 withheld water after 0, 2, 4, 6 and 8d, and one sample isolated from Lu-6 withheld water after 6d, were used for constructing cotton RNA-seq libraries which were sequenced at large-scale by ABlife Inc (Wuhan, China). Briefly, 10μg of total RNA of each sample was used for purifying polyadenylated mRNA by oligo(dT)-conjugated magnetic beads (Invitrogen). The purified mRNA was iron-fragmented at 95°C, followed by end repair and 5' adaptor ligation. Then, reverse transcription was performed with RT primer harboring 3' adaptor sequence, and randomized into hexamers. The cDNAs were purified and amplified by PCR. PCR products corresponding to 200 – 500 bp were purified, quantified and stored at –80°C until used for sequencing.

For high-throughput sequencing, the directional RNA-seq libraries were prepared according to the manufacturer’s instructions, and applied to illumina GAIIx system for 80 nt single-end sequencing by ABlife Inc (Wuhan, China) or to Hiseq 2000 system for 100 nt pair-end sequencing by BGI Inc (Shenzhen, China). These sequence data were deposited in NCBI Sequence Read Archive (SRA, http://www.ncbi.nlm.nih.gov/Traces/sra) with accession number SRP030288.

### Extraction of clean reads

Raw sequences were transformed into clean reads after certain steps of data processing, including removal of the 3’ adaptor sequence, low-quality reads, and reads that are too short (less than 20 nt), leaving clean reads to do the following analysis.

### Mapping of clean tag

The cotton sequencing data was compared with the upland cotton unigene data that has been published. *Arabidopsis* genes were used to annotate upland cotton unigenes, and total of 24159 unigenes could be noted.

### Analysis of differential expression genes

To predict gene function and calculate the functional category distribution frequency, Gene ontology (GO) analysis was employed. In the differential gene expression analysis, we applied the software edger which is a specific software for differential expression analysis of the genes from RNA-Seq data. To judge whether a gene is differential expression, the analysis results based on fold change (FC≥2 or FC≤-2) and P-value(P≤0.01).

### Data validation by quantitative RT-PCR analysis

Among the thousands of differentially expression genes, twelve genes with distinctly changed expression profiling were selected to conduct this experiment. Expression of the genes in leaves of cotton plants under drought stress was analyzed by real-time quantitative reverse transcription-polymerase chain reaction (RT-PCR) with the fluorescent intercalating dye SYBR-Green in a detection system (Opticon 2; MJ Research, Waltham, USA), using a cotton polyubiquitin gene (*GhUBI1*) as a standard control [26]. A two-step RT-PCR procedure was performed in the experiments. First, 2 μg of purified total RNA was reversely transcribed into cDNAs which were used as templates for PCR reactions using gene-specific primers (Table 5). Second, quantitative PCR was performed using PCR Master Mix (Toyobo, Osaka, Japan) according to the manufacturer’s instructions. Relative quantification of gene expression was determined using the comparative Ct method. To achieve optimal amplification, PCR conditions for each primer combination were optimized for annealing temperature, and PCR products were verified by melting curve analysis and confirmed on an agarose gel. Mean values and standard errors were calculated from three independent experiments with three biological replicates of cotton materials, and the data were normalized with the relative efficiency of each primer pair.

**Table 5:** Gene-specific primers used in real-time quantitative RT-PCR analysis.

Gene no.	primers
Contig6167	P1: 5’>GCAGAAGTTGCAAAGCTAAAAGAG<3’
	P2: 5’>CCTTATTCTACACTACAGGCTACA<3’
Contig11689	P1: 5’>GATGATAGTGCCATAAGGACGGAT<3’
	P2: 5’>TGATACATAGACATGCTTTCCCCC<3’
Contig6131	P1: 5’>TATCAAATGATACTGCATGCGGGG<3’
	P2: 5’>CACTACCAAGTCCCGACCAACTAA<3’
Contig3492	P1: 5’>GACAAACTCCCAGGACAGCATAAG<3’
	P2: 5’>GCAACCCCAAAAAGAAATACAGAG<3’
Contig5131	P1: 5’>GGTAAAGGATATTGGTGCAGATTG<3’
	P2: 5’>GCTGGGCATTTACTCTGTTGAGTA<3’
Contig6167	P1: 5’>ACAAAGCCAGCCACCGTAAACCCT<3’
	P2: 5’>TCTTCATTTTCCTCACAAGCAGGC<3’
Contig3187	P1: 5’>GCGATGCGTGGAGGACAAATCTAT<3’
	P2: 5’>CATCAGGCAGAGGACGAGACAA<3’
Contig12392	P1: 5’>TACTGTGGTGTGGGAGGTACGAGT<3’
	P2: 5’>CTTGTTCTTCTGCCCATCAGCATT<3’
Contig13443	P1: 5’>CCCAAGGACAAATAGTTCGATTCC<3’
	P2: 5’>GGGGAAAACAGTTCAGTGGAGATC<3’
Contig14123	P1: 5’>TTAGCAAGTACGCTCGAACTGTGC<3’
	P2: 5’>CAGCAACCTCAGTCAGTGAACCTT<3’
Contig14357	P1: 5’>AGAAGATGAGTGCCTTATCCTAGC<3’
	P2: 5’>GGCACCCTTTTGCCGTCATGTTTT<3’
Contig1444	P1: 5’>GGTGAGGACTTTCGAAACACATTG<3’
	P2: 5’>AATGCAGCAGCATTCGGACCATTC<3’

## Supporting Information

Table S1 **Go term analysis of differentially expressed genes between J-13 and Lu-6 6 days past drought treatment.**
(XLS)

Table S2 **Go term analysis of continually-differentially expressed genes.**
(XLS)

## References

[1] Turner NC, Wright GC, Siddique KHM (2001) Adaptation of grain legumes (pulses) to water-limited environments. Adv Agro 71: 193-231.

[2] Nguyen HT, Babu RC, Blum A (2007) Breeding for drought resistance in rice: physiology and molecular genetics considerations. Crop Sci 37: 1426-1434.

[3] Chen ZJ, Scheffler BE, Dennis E, Triplett BA, Zhang T et al. (2007) Toward sequencing cotton (*Gossypium*) genomes. Plant Physiol 145:1303-1310. PubMed: 18056866. 18056866 10.1104/pp.107.107672 PMC2151711

[4] Hendrix B, Stewart JM (2005) Estimation of the nuclear DNA content of Gossypium species. Ann Bot 95: 789-797. doi:10.1093/aob/mci078. PubMed: 15701660. 15701660 PMC4246727

[5] Truman CC, Strickland TC, Potter TL, Franklin DH, Bosch DD et al. (2007) Variable rainfall intensity and tillage effects on runoff, sediment, and carbon losses from a loamy sand under simulated rainfall. J Environ Qual 36: 1495-1502. doi:10.2134/jeq2006.0018. PubMed: 17766829. 17766829

[6] Bauer PJ, Fortnum BA, Frederick JR (2010) Cotton responses to tillage and rotation during the turn of the century drought. Agron J 102: 1145-1148. doi:10.2134/agronj2010.0037.

[7] Chaves MM, Oliveira MM (2004) Mechanisms underlying plant resilience to water deficits: prospects for water-saving agriculture. J Exp Bot 55: 2365-2384. doi:10.1093/jxb/erh269. PubMed: 15475377. 15475377

[8] Stiller WN, Read JJ, Constable GA, Reid PE (2005) Selection for water use efficiency traits in a cotton breeding program: cultivar differences. Crop Sci 45: 1107-1113. doi:10.2135/cropsci2004.0545.

[9] Campbell BT, Chee PW, Lubbers E, Bowman DT, Meredith WR et al. (2011) Genetic improvement of the Pee Dee cotton germplasm collection following seventy years of plant breeding. Crop Sci 51: 955-968. doi:10.2135/cropsci2010.09.0545.

[10] He ZP, Zhou QQ, Zhu WJ (2001) Higher lint percent and drought tolerant cotton line selected by radiation breeding. Shi Yan Sheng Wu Xue Bao 34: 51-54 (in Chinese). PubMed: 12549010. 12549010

[11] Guo YH, Yu YP, Wang D, Wu CA, Yang GD et al. (2009) GhZFP1, a novel CCCH-type zinc finger protein from cotton, enhances salt stress tolerance and fungal disease resistance in transgenic tobacco by interacting with GZIRD21A and GZIPR5. New Phytol 183: 62-75. doi:10.1111/j.1469-8137.2009.02838.x. PubMed: 19402879. 19402879

[12] Zhang L, Xi D, Li S, Gao Z, Zhao S et al. (2011) A cotton group C MAP kinase gene, GhMPK2, positively regulates salt and drought tolerance in tobacco. Plant Mol Biol 77: 17-31. doi:10.1007/s11103-011-9788-7. PubMed: 21590508. 21590508

[13] Zhang L, Li Y, Lu W, Meng F, Wu CA et al. (2012) Cotton GhMKK5 affects disease resistance, induces HR-like cell death, and reduces the tolerance to salt and drought stress in transgenic *Nicotiana* *benthamiana* . J Exp Bot 63: 3935-3951. doi:10.1093/jxb/ers086. PubMed: 22442420. 22442420 PMC3388830

[14] Gao SQ, Chen M, Xia LQ, Xiu HJ, Xu ZS et al. (2009) A cotton (*Gossypium* *hirsutum*) DRE-binding transcription factor gene, GhDREB, confers enhanced tolerance to drought, high salt, and freezing stresses in transgenic wheat. Plant Cell Rep 28: 301-311. doi:10.1007/s00299-008-0623-9. PubMed: 19005655. 19005655

[15] Shi J, Zhang L, An H, Wu C, Guo X (2011) GhMPK16, a novel stress-responsive group D MAPK gene from cotton, is involved in disease resistance and drought sensitivity. BMC Mol Biol 12: 22. doi:10.1186/1471-2199-12-22. PubMed: 21575189. 21575189 PMC3117701

[16] Sun G, Xie F, Zhang B (2011) Transcriptome-wide identification and stress properties of the 14-3-3 gene family in cotton (*Gossypium* *hirsutum* L.). Funct Integr Genomics 11: 627-636. 10.1007/s10142-011-0242-3 21805362

[17] Padmalatha KV, Dhandapani G, Kanakachari M, Kumar S, Dass A et al. (2012) Genome-wide transcriptomic analysis of cotton under drought stress reveal significant down-regulation of genes and pathways involved in fibre elongation and up-regulation of defense responsive genes. Plant Mol Biol 78: 223-246. doi:10.1007/s11103-011-9857-y. PubMed: 22143977. 22143977

[18] Ranjan A, Nigam D, Asif MH, Singh R, Ranjan S et al. (2012) Genome wide expression profiling of two accession of *G*. *herbaceum* L. in response to drought. BMC Genomics 13: 94. 22424186 10.1186/1471-2164-13-94 PMC3320563

[19] Taliercio E, Allen RD, Essenberg M, Klueva N, Nguyen H et al. (2006) Analysis of ESTs from multiple *Gossypium* *hirsutum* tissues and identification of SSRs. Genome 49: 306-319. doi:10.1139/G05-115. PubMed: 16699550. 16699550

[20] Wang K, Wang Z, Li F, Ye W, Wang J et al. (2012) The draft genome of a diploid cotton *Gossypium* *raimondii* . Nat Genet 44: 1098-1103. doi:10.1038/ng.2371. PubMed: 22922876. 22922876

[21] Park W, Scheffler BE, Bauer PJ, Campbell BT (2012) Genome-wide identification of differentially expressed genes under water deficit stress in upland cotton (*Gossypium* *hirsutum* L.). BMC Plant Biology 12: 90. 22703539 10.1186/1471-2229-12-90 PMC3438127

[22] Chance B, Maehly AC (1955) Assay of catalase and peroxidase. Methods Enzymol 2: 764-775. doi:10.1016/S0076-6879(55)02300-8.

[23] Nouchi I (1993) Changes in antioxidant levels and activities of related enzymes in rice leaves exposed to ozone. Soil Sci Plant Nutr 39: 309-320. doi:10.1080/00380768.1993.10417002.

[24] Heath RL, Packer L (1968) Photoperoxidation in isolated chloroplasts. I. Kinetics and stoichiometry of fatty acid peroxidation. Arch Biochem Biophys 125: 189-198. doi:10.1016/0003-9861(68)90654-1. PubMed: 5655425. 5655425

[25] Bajji M, Kinet J, Lutts S (2002) The use of the electrolyte leakage method for assessing cell membrane stability as a water stress tolerance test in durum wheat. Plant Growth Regul 36: 61-70. doi:10.1023/A:1014732714549.

[26] Li XB, Fan XP, Wang XL, Cai L, Yang WC (2005) The cotton ACTIN1 gene is functionally expressed in fibers and participates in fiber elongation. Plant Cell 17: 859-875. doi:10.1105/tpc.104.029629. PubMed: 15722467. 15722467 PMC1069704

[27] Zhang XY, Liu CL, Wang JJ, Li FG, Ye WE (2007) Evaluation to the drought tolerance of cotton by PEG water-stress. J Cotton Sci 19: 205- 209.

[28] Alejandro P (2010) Cotton: Review of World Situation. International Advisory Committee 63(3)

[29] Zhang B, Wang Q, Wang K, Pan X, Liu F et al. (2007) Identification of cotton microRNAs and their targets. Gene 397: 26–37. doi:10.1016/j.gene.2007.03.020. PubMed: 17574351. 17574351

[30] Knox JP, Dodge AD (1985) Singlet oxygen and plants. Phytochemistry 24: 889-893. doi:10.1016/S0031-9422(00)83147-7.

[31] McKersie BD, Leshem YY (1994) Stress and stress coping in cultivated plants. Dordrecht: Kluwer Publishing House pp. 15-54.

[32] Solecka D (1997) Role of phenylpropanoid compounds in plant responses to different stress factors. Acta Physiol Plant 19: 257-268. doi:10.1007/s11738-997-0001-1.

[33] Dai M, Xue Q, McCray T, Margavage K, Chen F et al. (2013) The PP6 phosphatase regulates ABI5 phosphorylation and abscisic acid signaling in Arabidopsis. Plant Cell 25: 517-534. doi:10.1105/tpc.112.105767. PubMed: 23404889. 23404889 PMC3608775

[34] Miura K, Lee J, Jin JB, Yoo CY, Miura T et al. (2009) Sumoylation of ABI5 by the Arabidopsis SUMO E3 ligase SIZ1 negatively regulates abscisic acid signaling. Proc Natl Acad Sci USA 106: 5418-5423. PubMed: 19276109. 19276109 10.1073/pnas.0811088106 PMC2664011

[35] Lopez-Molina L, Mongrand S, Chua NH (2001) A postgermination developmental arrest checkpoint is mediated by abscisic acid and requires the ABI5 transcription factor in Arabidopsis. Proc Natl Acad Sci USA 98: 4782-4787. PubMed: 11287670. 11287670 10.1073/pnas.081594298 PMC31911

[36] Hare PD, Cress WA (1997) Metabolic implications of stress-induced proline accumulation in plants. Plant Growth Regul 21: 79-102. doi:10.1023/A:1005703923347.

[37] Rhodes D, Handa S, Bressan RA (1986) Metabolic changes associated with adaptation of plant-cells to water-stress. Plant Physiol 82: 890-903. doi:10.1104/pp.82.4.890. PubMed: 16665163. 16665163 PMC1056230

[38] Verbruggen N, Hermans C (2008) Proline accumulation in plants: a review. Amino Acids 35: 753-759. doi:10.1007/s00726-008-0061-6. PubMed: 18379856. 18379856

[39] Szabados L, Savouré A (2010) Proline: a multifunctional amino acid. Trends Plant Sci 15: 89-97. doi:10.1016/j.tplants.2009.11.009. PubMed: 20036181. 20036181

[40] Wilkinson S, Davies WJ (2010) Drought, ozone, ABA and ethylene. New insights from cell to plant to community. Plant Cell Environ 33: 510-525. doi:10.1111/j.1365-3040.2009.02052.x. PubMed: 19843256. 19843256

[41] Munns R, Sharp RE (1993) Involvement of abscisic-acid in controlling plant-growth in soils of low water potential. Aust J Plant Physiol 20: 425-437. doi:10.1071/PP9930425.

[42] Hose E, Steudle E, Hartung W (2000) Abscisic acid and hydraulic conductivity of maize roots: a study using cell- and rootpressure probes. Planta 211: 874-882. 11144273 10.1007/s004250000412

[43] Parent B, Hachez C, Redondo E, Simonneau T, Chaumont F et al. (2009) Drought and abscisic acid effects on aquaporin content translate into changes in hydraulic conductivity and leaf growth rate. A trans-scale approach. Plant Physiol 149: 2000-2012. doi:10.1104/pp.108.130682. PubMed: 19211703. 19211703 PMC2663730

[44] Abeles FB, Morgan PW, Saltveit ME (1992) Ethylene in Plant Biology, 2nd ed. San Diego: Academic Press.

[45] Atkinson CJ, Davies WJ, Mansfield TA (1989) Changes in stomatal conductance in intact aging wheat leaves in response to abscisic-acid. J Exp Bot 218: 1021-1028.

[46] Feys BJ, Parker JE (2000) Interplay of signaling pathway in plant disease resistance. Trends Genet 16: 449-455. doi:10.1016/S0168-9525(00)02107-7. PubMed: 11050331. 11050331

[47] Glazebrook J (2001) Genes controlling expression of defense responses in Arabidopsis - 2001 status. Curr Opin Plant Biol 4: 301-308. doi:10.1016/S1369-5266(00)00177-1. PubMed: 11418339. 11418339

